# A Case of De Novo Membranous Nephropathy Causing Renal Transplant Rejection

**DOI:** 10.7759/cureus.26246

**Published:** 2022-06-23

**Authors:** Sarah C Kurkowski, Michael J Thimmesch, Amro Abdelghani, Yasir H Abdelgadir

**Affiliations:** 1 Medical School, Medical College of Wisconsin, Milwaukee, USA; 2 Internal Medicine, Medical College of Wisconsin, Milwaukee, USA; 3 Department of Medicine, Medical College of Wisconsin, Milwaukee, USA

**Keywords:** post-transplant membranous nephropathy, lupus nephritis, renal transplant rejection, acute transplant rejection, de novo membranous nephropathy

## Abstract

We present a novel case of de novo membranous nephropathy (DNMN) leading to transplant rejection in a 51-year-old female patient. The patient has a transplant history of two renal transplants for end-stage renal disease due to lupus nephritis. She had a prior unrelated, living donor kidney transplant that was subsequently replaced by a deceased donor kidney transplant due to graft failure. This patient’s case is intriguing because DNMN is a rare cause of transplant rejection, and the literature demonstrates a scarcity of clinical examples. Interestingly, post-transplant DNMN has been suggested to be a separate disease from recurrent post-transplant MN and is associated with separate risk factors and diagnostic findings. As DNMN is considered a manifestation of antibody-mediated rejection, it should be treated with immunosuppressive therapy. As such, the presented case has received immunosuppressive therapy. In addition, DNMN is associated with humoral alloimmunity. Potentially other inflammatory processes (such as infection/potential UTI in our patient’s case) could cause exposure to undetectable donor antigens on renal transplants leading to antibody-mediated rejection via DNMN.

## Introduction

Membranous nephropathy (MN) is known to be the most common cause of nephrotic syndrome [1]. Of the cases of MN, the vast majority (80%) are confined to the kidneys and thus called primary membranous nephropathy (PMN) [1]. The remaining 20% of MN cases arise secondary to other processes, such as infections (hepatitis C virus, hepatitis B virus, human immunodeficiency virus, parasitic organisms), malignancy (solid tumors such as lung or prostate, non-Hodgkin lymphoma, plasma cell dyscrasias, chronic lymphocytic leukemia), autoimmune diseases (systemic lupus erythematosus, thyroiditis, rheumatoid arthritis, antineutrophil cytoplasmic antibody-associated vasculitis, anti-glomerular basement membrane disease, IgG4 vasculitis), alloimmune disease (graft versus host disease, de novo membranous nephropathy in transplant), drugs/toxins (non-steroidal anti-inflammatory drugs, cyclooxygenase-2 inhibitors, penicillamine, gold), and diabetes [1]. PMN is highly associated with phospholipase A2 receptor (PLA2R) IgG4 antibodies (seen in serum or on biopsy) or thrombospondin domain-containing 7A (THSD7A) antibodies in serum. 70% of cases identify PLA2R antibodies in the serum, 15% find PLA2R antibodies via biopsy, and 3-5% identify THSD7A antibodies in serum. About 10% of cases of PMN do not have either of these antibodies identified, yet it has been proposed that another unknown anti-podocyte antibody is the cause [1]. PMN can be subdivided into de novo (DNMN) and recurrent membranous nephropathy (RMN), both of which can cause nephrotic syndrome in patients after renal transplantation. MN in transplant recipients also increases the risk of allograft loss [2].

Nephrotic syndrome is characterized by the loss of 3 grams of protein, or more, per day in the urine. Serum albumin levels are often low as well, due to loss of albumin in the urine (usually serum albumin is < 2.5 g/dL in nephrotic syndrome). Cholesterol and triglyceride levels are increased in typical nephrotic syndrome. While blood creatinine levels are measured to assess renal function, they may not always be elevated at beginning of the disease as the degree of renal impairment varies between patients. To detect kidney damage from MN, ultrasound is used and would typically show increased renal echogenicity, which indicates intrarenal fibrosis [3]. A renal tissue biopsy can be obtained in more severe or complicated cases, as in the case we present in this article.

A previous study reported that the specific percent incidence of DNMN is difficult to obtain since transplant centers (both in the United States and worldwide) have differing indications for graft biopsy [4]. A retrospective study looking at 614 renal allograft transplant biopsies (between 1989 and 2006) found that only 11 (1.8%) patients had DNMN [5]. DNMN has been associated with specifically antibody-mediated transplant rejection and donor-specific antibodies in renal transplant patients [6, 7, 8]. A case series following 1550 renal transplant recipients in seven renal transplant centers throughout Paris, France, found that the event rate of DNMN in renal transplant patients was 1.9%. Of the 1550 renal transplant patients, 1000 had renal graft biopsies taken. Nineteen of the 1000 biopsies showed DNMN, leading to the 1.9% event rate. Surprisingly, among this cohort, the authors did not find an association between DNMN and the patient age, sex, donor-recipient HLA phenotype, graft number (1st vs 2nd), number of previous rejection episodes, number or length of acute tubular necrosis events, nor viral/bacterial infections [9]. Though, other more recent case reports propose that DNMN is more commonly seen in patients with hepatitis C virus infection [7]. In contrast to primary RMN, DNMN is highly associated with IgG1 autoantibodies [10].

## Case presentation

A 51-year-old female presented to the emergency department following the finding of an elevated creatinine level during her belatacept infusion appointment. Her past medical history was significant for end-stage renal disease (due to lupus nephritis) status post two renal transplants, systemic lupus erythematosus (SLE) with anticardiolipin antibody syndrome on warfarin, and fixed patent foramen ovale with previous cerebrovascular accident complicated by right-sided weakness.

The patient’s baseline creatine was around 1.7 mg/dL. At admission, the creatinine level was 2.75 mg/dL, and had been worsening over the past month (the in-between two creatinine levels were 2.48 mg/dL and 2.61 mg/dL). The urine protein/creatinine ratio was elevated at 2.10 on admission. The estimated glomerular filtration rate (GFR) was low (18 mL/min/1.73 sqm) compared to the previous month (around 30 mL/min/1.73 sqm).

She denied any new symptoms over the preceding days/week and had not noticed any changes in urine output. She denied nausea, vomiting, and diarrhea. The initial lab work on admission (which did not include lupus serological markers) was largely unremarkable outside of possible urinary tract infection detected on urinalysis. Due to concern for possible UTI, the patient was started on amoxicillin-clavulanate but discontinued two days later when the culture grew normal flora.

The patient underwent renal transplant 18 years and five years before current hospital admission. She received a living-unrelated kidney transplant in 2004. Her transplant was failing, and she received a second transplant, which was a deceased donor kidney transplant, in 2017. She was not on dialysis prior to transplantation. Her pretransplant panel reactive antibody (PRA) was 61% for class I and 92% for class II. She received a transplant with an HLA A/B-1 and DR-1 antigen match. Cytomegalovirus (CMV) status at transplant was D+R- (donor positive, recipient negative). She received thymoglobulin for induction at that time. Since the second kidney transplant five years ago, she had been hospitalized with a ruptured colonic diverticulum requiring emergency colectomy and an acute kidney injury (AKI) related to rhabdomyolysis.

**Figure 1:**
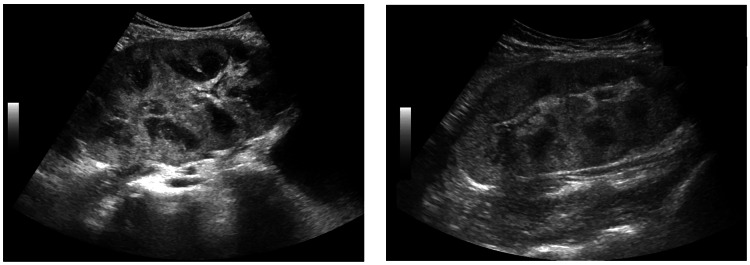
Sagittal views of transplanted kidney on ultrasound showing increased renal echogenicity

A kidney ultrasound was performed and did not show signs of renal artery stenosis, but did show increased echogenicity (Figure 1), which could indicate transplant rejection. The Doppler ultrasound showed benign findings: two transplant main renal arteries patent with a sharp systolic upstroke and continuous diastolic flow with no perivascular tissue vibration. The main renal arteries peak velocities were 119.0 cm/sec and 125.4 cm/sec. The transplant intrarenal arteries were patent with no tardus parvus or high resistance waveforms. The resistive index was 0.70-0.72 (normal is < 0.8). The pulsatility index was 1.36-1.46 (normal is <1.8). The transplant renal vein, ipsilateral iliac artery, and vein were also patent with normal cardiac phasicity. The patient’s increasing creatinine levels ultimately prompted admission for renal biopsy to determine the etiology of her deteriorating renal function. At the time of admission, the patient’s immunosuppression regimen consisted of cyclosporine (50 mg capsule two times daily), belatacept (300 mg IV infusion every four weeks), mycophenolate (250 mg capsule two times daily), and prednisone (5 mg tablet daily).

An ultrasound-guided kidney biopsy was performed. The biopsy revealed antibody-mediated rejection and showed glomerular immune complex deposition, consistent with de novo post-transplant MN. It also revealed moderate microvascular inflammation with focal C4d deposition, suspicious for active antibody-mediated rejection. Immunofluorescence revealed finely granular capillary wall staining for IgG, C3, kappa light chains, and lambda light chains. The specimen was found to be IgG1 predominant. This supported the diagnosis of de novo post-transplant MN. Other potential diagnoses on the differential were primary RMN and membranous lupus nephritis. Primary RMN was unlikely as IgG4 staining would have been expected. The absence of "full-house" staining (all five major immunofluorescent stains on a renal biopsy (IgM, IgG, IgA, C3, and C1q) are positive) or significant IgG2 deposition was against the diagnosis of membranous lupus nephritis.

During her hospital course, a right internal jugular catheter was placed. She received four doses of high-intensity steroids (250 mg IV methylprednisolone), which somewhat improved her GFR (increased to 22 mL/min/1.73 sqm from 18 mL/min/1.73 sqm). Her creatinine during the hospital course peaked at 2.86 mg/dL, but after steroids, it decreased to 2.33 mg/dL. She was started on plasmapheresis as well during her stay and received three total rounds of plasmapheresis followed by 10 grams of intravenous immune globulin (IVIG). She received one dose (600 mg IV) of rituximab.

After the three plasmapheresis sessions, her creatinine and GFR appeared to respond well to treatment. GFR increased to 24 mL/min/1.73 sqm and creatinine decreased to 2.18 mg/dL. She was discharged home with instructions to follow up with outpatient transplant nephrology, complete three more sessions of plasmapheresis/IVIG (200 mg/kg), and one more infusion of 375 mg/m² rituximab. Plasmapheresis was used as a means of removing antibodies that may have led to the patient's acute transplant rejection.

During the next three months post-discharge, our patient’s creatinine levels fluctuated between 2.53 mg/dL and 2.20 mg/dL. The most recent creatinine level was 2.28 mg/dL, still not at her previous baseline. However, this may be her new baseline after the potential renal damage from DNMN. In addition, her urine protein/creatinine ratio was not significantly improving despite treatment: 1.81 three months post-discharge compared to 2.10 at admission.

## Discussion

Acute renal transplant rejection caused by DNMN is quite rare, as stated by multiple articles in which the event rate ranged from 1.8%-1.9% [5, 9]. While PMN is vastly and widely studied and understood, DNMN causing transplant rejection is understudied and not well-understood. PMN is associated with IgG4 staining on renal biopsy, while DNMN is associated with IgG1 staining [10]. DNMN is also associated with findings of antibody-mediated rejection on renal biopsy, evidenced by C4d deposition in peritubular capillaries [11]. In a retrospective case series, published in 2020, authors compared RMN and DNMN. DNMN was more likely to have concurrent antibody-mediated rejection and lower allograft survival when compared to RMN [12]. The same article also posed the conclusion that DNMN and RNM were likely two distinct diseases, even though both are considered PMN [12]. DNMN is associated with humoral alloimmunity (a major cause of transplant failure) [12].

At the microscopic level, DNMN and RMN show similarities, yet the former is far less common. DNMN varies widely in both its presentation and course of disease, and can develop in transplant recipients who already have underlying inflammatory processes or diseases such as viral hepatitis (Hepatitis C virus), Alport syndrome, post-renal obstruction, and renal infarction, or in combination with IgA nephritis. The onset of DNMN also shows a correlation to the histologic signs of allograft rejection, suggesting that DNMN may have been induced by donor antibodies. However, one study found that DNMN still developed in recipients of “full house” HLA-matched kidneys [8]. The correlation noted earlier between DNMN and present underlying infection/disease may indicate that DNMN is a byproduct of an already inflammatory environment. Such an environment could expose hidden antigens not previously identified in HLA-matching. This would then lead to the production of circulating antibodies, in situ formation of immune complexes, and the MN lesion [8]. At presentation, our patient had a potential urinary tract infection and was started on amoxicillin-clavulanate. While her urine culture grew normal flora and ultimately amoxicillin-clavulanate was discontinued, this does not completely rule out the possibility that she had a previous urinary tract infection. In addition, what initially led to her requiring a renal transplant was lupus nephritis from SLE, a well-known autoimmune inflammatory disease. Either a urinary tract infection or her chronic SLE could have spurred the development of DNMN.

Our patient’s case presented similarly to the few case reports of de novo post-transplant MN found in the literature. Diagnosis by antibody staining highlighted immunoglobulin G (IgG)1 as predominant in our patient; typical of DNMN [10]. IgG2 is the most commonly expressed antibody in lupus nephritis, however, our patient was absent of significant IgG2 deposition [13]. In one case series of four patients with DNMN post-transplant, the disease was associated with antibody-mediated rejection and de novo donor-specific class I and II antibodies. These four patients were diagnosed with DNMN 10-92 months post-transplant [6]. Our patient was diagnosed with DNMN and antibody-mediated rejection 60 months after her most recent kidney transplant. A second case report described a patient who underwent deceased donor kidney transplantation twice (unknown if donor-specific) and was diagnosed via kidney biopsy with DNMN. The authors also stated that the incidence of DNMN was greater in patients with chronic HCV than in those with no infection (3.6% to 0.36%) [7]. The case we described here did not have acute or chronic hepatitis C viral infection. Though not necessary for the development of DNMN leading to transplant rejection, it certainly would have increased her risk. Our patient also presented with increased renal echogenicity on her ultrasound (Figure 1) which strongly suggested that underlying kidney disease was present, as was the case once confirmed on renal biopsy [14]. Her subsequent treatment for acute transplant rejection with corticosteroids was also the correct treatment according to the literature [15]. Since DNMN is assumed to be a manifestation of antibody-mediated rejection, the treatment should aim at suppressing the immune system [11]. Therefore, our patient was discharged with the addition of plasmapheresis/IVIG (200 mg/kg) and 375 mg/m² Rituximab to her pre-admission regimen of cyclosporine (50 mg capsule two times daily), belatacept (300 mg IV infusion every four weeks), mycophenolate (250 mg capsule two times daily), and prednisone (5 mg tablet daily).

This case should prompt clinicians to be more suspicious of DNMN in post-renal transplant patients and not to eliminate the possibility of the disease/cause based on its rarity.

## Conclusions

DNMN is considered a manifestation of antibody-mediated rejection and requires treatment with immunosuppressive therapy. The exact cause or inciting event is unknown in the development of DNMN. However, that does not nullify the drastic effects it has on the patient and their health. The case presented here urges physicians and healthcare providers to keep DNMN in mind when renal transplant patients present with signs and symptoms concerning acute transplant rejection. It should not be ruled out simply because of its rarity when compared to other causes of acute transplant rejection.
